# The STUN (STop UNhealthy) Alcohol Use Now trial: study protocol for an adaptive randomized trial on dissemination and implementation of screening and management of unhealthy alcohol use in primary care

**DOI:** 10.1186/s13063-021-05641-7

**Published:** 2021-11-16

**Authors:** Daniel E. Jonas, Colleen Barclay, Debbie Grammer, Chris Weathington, Sarah A. Birken, Darren A. DeWalt, Kimberly A. Shoenbill, Marcella H. Boynton, Monique Mackey, Sean Riley, Samuel Cykert

**Affiliations:** 1 Division of General Internal Medicine and Geriatrics, Department of Internal Medicine, The Ohio State University, 2050 Kenny Road, Columbus, Ohio 43221 USA; 2 Cecil G. Sheps Center for Health Services Research, CB 7590, University of North Carolina at Chapel Hill, Chapel Hill, NC 27599 USA; 3 North Carolina Area Health Education Centers, CB 7165, University of North Carolina at Chapel Hill, Chapel Hill, NC 27599 USA; 4 Department of Implementation Science, Wake Forest University School of Medicine, Winston-Salem, NC 27101 USA; 5 Division of General Medicine and Clinical Epidemiology, CB 7110, University of North Carolina at Chapel Hill, Chapel Hill, NC 27599 USA; 6 Department of Family Medicine, CB 7370, University of North Carolina at Chapel Hill, Chapel Hill, NC 27599 USA; 7 Program on Health and Clinical Informatics, CB 7064, University of North Carolina at Chapel Hill, Chapel Hill, NC 27599 USA

**Keywords:** Alcohol, Unhealthy alcohol use, Screening, Counseling, Practice facilitation, Embedded telehealth, Primary care, Implementation science, Quality improvement

## Abstract

**Background:**

Unhealthy alcohol use is a leading cause of preventable deaths in the USA and is associated with many societal and health problems. Less than a third of people who visit primary care providers in the USA are asked about or ever discuss alcohol use with a health professional.

**Methods/design:**

This study is an adaptive, randomized, controlled trial to evaluate the effect of primary care practice facilitation and telehealth services on evidence-based screening, counseling, and pharmacotherapy for unhealthy alcohol use in small-to-medium-sized primary care practices. Study participants will include primary care practices in North Carolina with 10 or fewer providers. All enrolled practices will receive a practice facilitation intervention that includes quality improvement (QI) coaching, electronic health record (EHR) support, training, and expert consultation. After 6 months, practices in the lower 50th percentile (based on performance) will be randomized to continued practice facilitation or provision of telehealth services plus ongoing facilitation for the next 6 months. Practices in the upper 50th percentile after the initial 6 months of intervention will continue to receive practice facilitation alone. The main outcome measures include the number (and %) of patients in the target population who are screened for unhealthy alcohol use, screen positive, and receive brief counseling. Additional measures include the number (and %) of patients who receive pharmacotherapy for AUD or are referred for AUD services. Sample size calculations determined that 35 practices are needed to detect a 10% increase in the main outcome (percent screened for unhealthy alcohol use) over 6 months.

**Discussion:**

A successful intervention would significantly reduce morbidity among adults from unhealthy alcohol use by increasing counseling and other treatment opportunities. The study will produce important evidence about the effect of practice facilitation on uptake of evidence-based screening, counseling, and pharmacotherapy for unhealthy alcohol use when delivered on a large scale to small and medium-sized practices. It will also generate scientific knowledge about whether embedded telehealth services can improve the use of evidence-based screening and interventions for practices with slower uptake. The results of this rigorously conducted evaluation are expected to have a positive impact by accelerating the dissemination and implementation of evidence related to unhealthy alcohol use into primary care practices.

**Trial registration:**

ClinicalTrials.govNCT04317989. Registered on March 23, 2020.

**Supplementary Information:**

The online version contains supplementary material available at 10.1186/s13063-021-05641-7.

## Contributions to the literature

Practice facilitation improves chronic disease care measures and adoption of evidence-based guidelines in primary care, but research is lacking on its effect on uptake of screening, counseling, and referral for unhealthy alcohol use.

Our study will evaluate whether practice facilitation can address and overcome key barriers to implementation (e.g., lack of a formal process) of these important services.

Our study will also evaluate the effect of embedded telehealth that connects patients with external counselors and services to overcome barriers to counseling in primary care, such as competing priorities, limited counseling experience and skills, and limited access to services.

## Background

Unhealthy alcohol use is the third leading cause of preventable deaths among working-age adults in the USA and is associated with many societal and health problems [1–3]. It is a key contributor to recent declines in US life expectancy, especially among middle-aged, Americans and those living in rural areas [4, 5]. National guidelines recommend no more than 4 drinks per day and 14 per week for men under 65, and no more than 3 drinks per day and 7 per week for all women and for men 65 and older [6, 7]. However, over 20% of primary care patients in the USA drink alcoholic beverages in excess of the recommended limits [8].

Multiple systematic reviews and recommendations have established that screening with brief validated questionnaires can accurately detect unhealthy alcohol use [9–11]. After detection, brief counseling interventions aim to reduce or eliminate risky drinking. Motivational interviewing techniques are commonly used in effective counseling interventions, and they can be an effective patient-centered approach for achieving behavior change [12, 13] Multiple systematic reviews have established the benefits of counseling (delivered by a variety of provider types) in primary care after screening, showing significant reductions in alcohol consumption [9–11]. For patients with alcohol use disorder (AUD), multiple more intensive treatment options are available; to date, no single intervention approach has been shown to be clearly superior to others in eliciting long-lasting reductions in unhealthy drinking. Twelve-step programs (e.g., alcoholics anonymous), cognitive behavior therapy, motivational enhancement therapy, and pharmacotherapy for AUD are among the commonly offered treatments [14].

Despite recommendations to screen for unhealthy alcohol use in the primary care setting, the burden of illness associated with it, and the existence of effective interventions, relatively few people who visit primary care providers in the USA are asked about alcohol use or ever discuss alcohol use with a health professional [15, 16]. A major barrier is that many practices often lack a formal process for screening and delivery of appropriate interventions [17, 18]. Additional barriers include competing priorities, limited skills in the delivery of counseling in primary care, and limited access to services (e.g., counseling for AUD) [19–21]. More than half of US adults have visits with primary care providers [22], and these professionals enjoy extraordinarily high levels of trust from the public [23]. Primary care visits represent an important opportunity to address unhealthy alcohol use by providing a safe environment to discuss the issue with a trusted and skilled health professional.

A growing body of evidence indicates that practice facilitation, an implementation science method that uses a team-based, QI approach to guide organizational change, [24] is an effective strategy for implementing evidence-based practices. Practice facilitation interventions in healthcare involve helping practices with workflow analysis and redesign, EHR support including building templates and decision support tools, evidence-based protocols, population health lists, data feedback (e.g., run charts) and benchmarking, standing orders, and the utilization of the Model for Improvement, an integrated approach to process improvement that delivers quick and substantial results in quality and productivity in diverse settings [25]. A systematic review of 23 studies found that primary care practices that received practice facilitation were more likely to adopt evidence-based guidelines relative to those who did not receive practice facilitation (OR 2.76, 95% CI, 2.18–3.43) [26]. A more recent 2018 systematic review of 25 randomized controlled trials (RCTs) and cohort studies found that practice facilitation improved a variety of chronic disease care measures, including those for cancer and cardiovascular disease [27]. None of the studies in either review focused on unhealthy alcohol use, indicating a gap in our knowledge on how to best help primary care practices address unhealthy alcohol use. Practice facilitation has the potential to address and overcome key barriers to implementation (e.g., lack of a formal process, limited counseling skills). However, some practices may lack the capacity to deliver counseling with their available resources and personnel. Such practices may benefit from having additional support through embedded telehealth services to connect their patients with external counselors and services, potentially overcoming barriers of competing demands and the lack of adequate skills to deliver effective counseling.

### Objectives and aims

In October 2019, the U.S. Department of Health and Human Services, through the Agency for Healthcare Research and Quality (AHRQ), launched an initiative to fund six research studies intended to help hundreds of primary care practices screen for and reduce unhealthy alcohol use in primary care [28]. This protocol describes one of those studies. The STop UNhealthy (STUN) Alcohol Use Now study is an adaptive RCT that will be conducted in small and medium-sized primary care practices (10 providers or fewer) across North Carolina. The study aims to evaluate the effect of primary care practice facilitation on (1) uptake of evidence-based screening and brief intervention (SBI) for unhealthy alcohol use and (2) uptake of evidence-based counseling and pharmacotherapy for alcohol use disorder (AUD). For practices with slower uptake of SBI, the study will evaluate the effect of using telehealth services to deliver SBI for unhealthy alcohol use, and counseling and pharmacotherapy for AUD. Finally, the study will evaluate the effect of practice facilitation on the implementation of clinical practice and office systems changes to improve SBI and pharmacotherapy for AUD. Our primary hypotheses are that practice facilitation (and, in the case of slow-uptake practices, the further addition of telehealth) will improve processes of care for unhealthy alcohol use and increase rates of screening, identification, and appropriate interventions.

## Methods/design

### Study setting

North Carolina (NC) is the 10th largest state in the USA with a population of over 10 million and a racial and ethnic composition of 71% White, 22% Black, and 9.6% Latino, respectively [29]. The burden of unhealthy alcohol use in NC remains large, accounting for an estimated 4000 deaths annually as well as substantial morbidity [30]. Approximately, 17% of adults in North Carolina engage in unhealthy alcohol use and 1 in 11 deaths among working-age adults (20–64 years old) in North Carolina are attributable to alcohol [31, 32]. The most common cause of alcohol-related deaths in NC is alcohol-impaired driving fatalities [33].

About 7400 physicians and 2000 advanced practice providers work mainly as primary care providers in North Carolina, according to 2019 NC licensure data in the North Carolina Health Professions Data System [34]. Practice size estimates vary according to practice definition (tax ID# vs. single geographic location), but based on the provider to practice ratios, we estimate that there are approximately 2200 primary care practices in NC. Thirty to 40% of these remain unaffiliated with larger health systems, and almost all have fully implemented EHRs. The practices eligible for enrollment in STUN are skewed toward rural areas.

### Research design

**Fig. 1:**
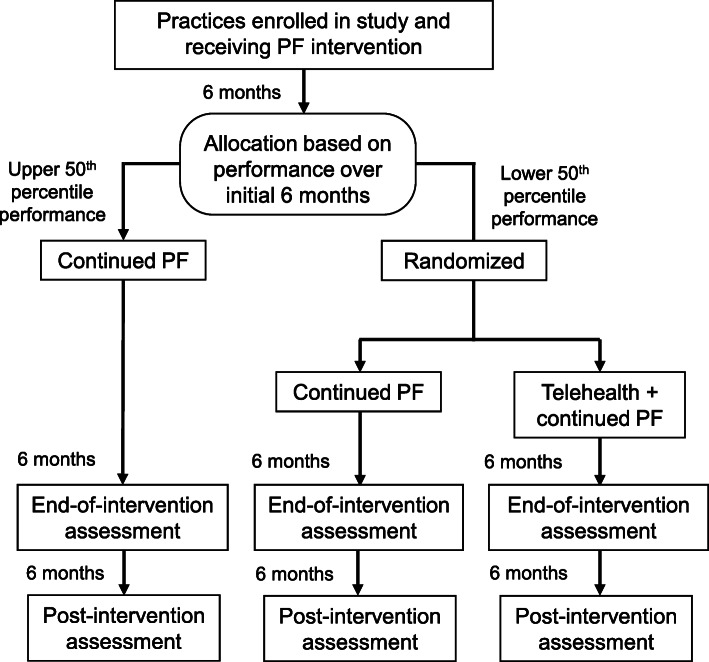
Study flow diagram

STUN Alcohol Use Now is an adaptive, randomized, controlled trial to evaluate the effect of primary care practice facilitation and the effect of using telehealth services on evidence-based screening, counseling, and pharmacotherapy for AUD (Fig. 1).

**Fig. 2:**
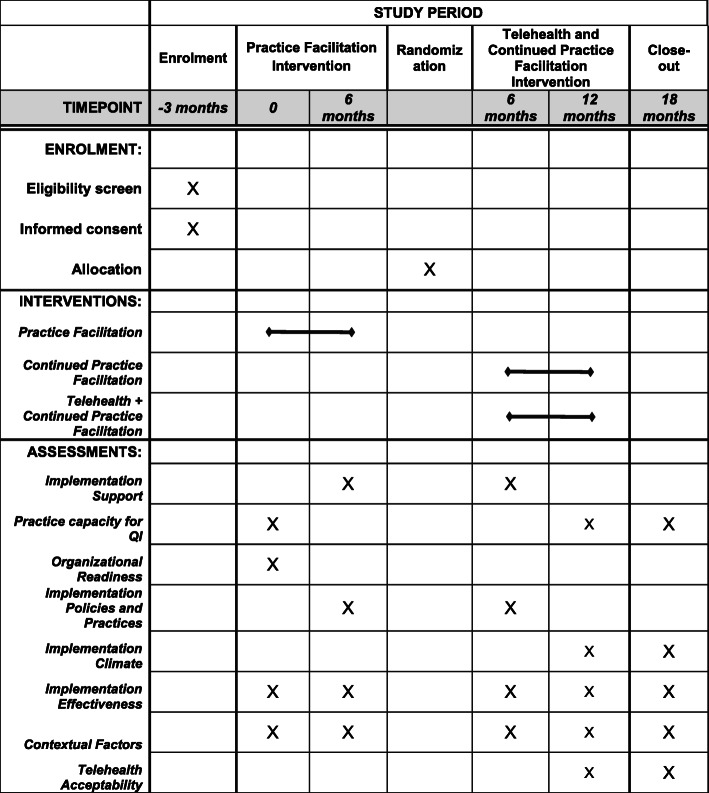
SPIRIT figure: Schedule of enrolment, interventions, and assessments

The design is considered an adaptive trial design because inclusion in the randomized portion of the study is based on practice performance during the initial 6 months of the intervention. All enrolled practices will receive the practice facilitation intervention. We followed the SPIRIT guidance for reporting the content of this study protocol and completed the SPIRIT checklist (supplemental file) and the SPIRIT figure (Fig. 2) [35].

After practices have received the practice facilitation intervention for 6 months, those in the lower 50th percentile of performance will be block randomized to continued practice facilitation or to using telehealth services plus continued practice facilitation for the next 6 months. The random allocation sequence will be generated using Microsoft excel by a data analyst who is not involved in recruitment or practice facilitation. The random allocation sequence will be stored in a password-protected electronic file and concealed from all other team members involved in recruitment and practice facilitation until after a practice has been determined to be in the lower 50th percentile of performance and has received the practice facilitation intervention for 6 months. The performance assessment over the initial 6 months will be based on the percentage of patients screened for unhealthy alcohol use and the percentage of patients who receive brief counseling when it is indicated (after screening indicates unhealthy alcohol use). Randomizing the lower 50th percentile will allow us to assess (1) whether the provision of telehealth services accelerates uptake for practices with slower uptake and (2) whether “staying the course” with ongoing practice facilitation is an effective strategy for those with slower uptake (comparing whether they catch up to the upper 50th percentile). Practices in the upper 50th percentile over the initial 6 months will continue to receive the practice facilitation intervention for another 6 months.

### Practice recruitment

Practice recruitment will be conducted by the practice facilitators (i.e., practice coaches). Due to COVID-19-related constraints, recruitment will be largely conducted on a virtual basis using phone calls, emails, and video conferencing. For the purpose of planning, guiding recruitment activities, and refining recruitment strategies, the research team will create and provide informational materials, schedule informational webinars with potential practice representatives, and participate in regular educational sessions with the practice facilitators. Based on enrollment targets set by the funder, hoping to reach and help as many practices as might be feasible, we initially aimed to enroll up to 135 small to medium-sized primary care practices. Our sample size calculations indicated that many fewer practices are needed to assess the effect of the intervention on our main outcomes. Specifically, 35 practices would be needed to detect a 10% increase in screening for Aim 1, and fewer practices would be needed if the magnitude of the increase in screening after the intervention is larger than 10% (as detailed in the *Statistical Power* section under Aim 1). Competing demands and constraints due to the pandemic tempered enrollment, leading the research team to adjust the enrollment targets to those based on our sample size calculations. Practices are eligible for enrollment if 10 or fewer providers occupy a single location and do not receive facilitation services specifically related to unhealthy alcohol use. Enrolling practices agree to the following: (1) work with practice facilitators for 4 to 8 h a month to implement an evidence-based screening process as well as a process for counseling and/or referring patients with unhealthy alcohol use, (2) participate in webinars conducted by project personnel about the screening and brief counseling process as well as how and when to prescribe medications for AUD, (3) respond to surveys about the practice environment and the improvement process, and (4) collect implementation effectiveness data on a monthly basis with help from practice facilitators.

Potential barriers to practice recruitment include fear of financial risk, dedicating practice resources to other new activities such as Medicaid reform or Accountable Care Organization participation to the exclusion of participation in this study, as well as concerns related to staffing issues, competing priorities, and lack of time. These barriers have been compounded during the COVID-19 pandemic as practices struggle to meet the demands of caring for patients in a changed environment and contend with new financial pressures arising from reduced patient visits. Practice facilitation will attempt to mitigate these barriers by helping practices adapt—for example, by streamlining workflows. During recruitment, we will also emphasize current evidence that the increase in social isolation, household pressures, and economic stress during the pandemic has been associated with an increase in unhealthy alcohol use [36–38].

### Practice support intervention

The NC Area Health Education Centers (AHEC) program has the permanent statewide infrastructure and highly trained personnel to support and deliver practice facilitation services and has developed strong relationships with primary care practices [39]. Participating practices will receive direct practice facilitation over the 12-month intervention period from NC AHEC personnel. The facilitator will ensure that key drivers for improvement are identified and that the practice is comfortable with implementing the improvement with rapid-cycle tests of change. Practices will receive 1–2 h of direct practice facilitation services per month and be expected to apply tests of change using a Plan-Do-Study-Act (PDSA) approach [40] fairly independently, confirmed and coached by a member of the facilitation team. The facilitator will ensure that the practice has established specific workflows for the unhealthy alcohol use measures and conduct periodic data checks to ascertain progress, sharing the results of these data checks with the practice (in an audit and feedback fashion). Webinars and video recordings will serve as additional tools that facilitators can use to educate practices on best practices for alcohol screening, counseling, and interventions. Expert consultation with physician faculty will be available, primarily virtually, to supplement facilitation efforts. While practice facilitation was initially planned as a combination of face-to-face meetings, phone calls, web-based video meetings, and email communication, remote communications will be the primary communication modality until risks from the pandemic have subsided. Facilitation meetings will emphasize the implementation of evidence-based protocols and the use of clinical algorithms by the following:

1. Forming clinical QI teams to engage the practice (or its participating clinicians) in a high standard of care delivery, including the use of standing orders, EHR templates, and clinical decision support tools.
2. Establishing human workflows including team-based roles to use these practice tools
3. Optimizing the use of the EHR to perform monthly data pulls to guide and evaluate the progress of the screening process, counseling, pharmacologic treatment, and associated referrals.
4. Developing spreadsheet registries and other electronic tools for practices lacking the EHR capabilities to develop the data resources described above.
5. Assisting the practices in optimizing billing for reimbursable SBI services.
6. Working with practices to develop proactive assigned roles and responsibilities to prepare the clinical team to develop needed care and engage patients.
7. Providing lists of available counseling and referral resources by region and county, to be used when primary care clinicians encounter patients whose unhealthy alcohol use, AUD, or comorbid behavior health conditions exceed the clinician’s comfort level or expertise.
8. For practices randomized to the telehealth group, protocols for scheduling and utilizing telehealth services will be developed.

The practice facilitator workforce is shovel-ready, with facilitators trained in 49 coaching competencies and training content continually updated to keep facilitators prepared to help practices respond to emergent needs in the field. They have prior experience working with practices on multiple topics. Upon enrollment, the facilitator establishes an intervention start date with the practice, taking into consideration current and upcoming conditions and priorities at the practice. Early tasks include assessing baseline alcohol screening rates and determining EHR reporting capabilities. The 12-month intervention period begins when the facilitator begins to work regularly with the practice on the processes described above.

### Evaluation framework

Implementation of practice facilitation to support the uptake of an evidence-based screening, brief counseling intervention, and referral to alcohol treatment represents a major innovation for addressing the substantial health burden of unhealthy alcohol use. Briefly, effective implementation is a function of the implementation support the practice receives and the policies and practices it employs to support innovation use.

Organized QI effort and capability will be the key driver of improvement, creating an environment in which primary care practices can embrace the implementation of the Chronic Care Model [41]). The Chronic Care Model emphasizes that practices use clinical decision support, clinical information systems, optimal delivery system design, self-management support, and community linkages to create prepared and proactive care teams and informed and motivated patients, leading to improved health outcomes [42]. The elements of the model fit well with the implementation of SBI for unhealthy alcohol use and pharmacotherapy for AUD.

Our primary hypotheses, that practice facilitation (and, in the case of slow-uptake practices, the further addition of telehealth), will improve processes of care for unhealthy alcohol use are motivated by adult learning theory and social cognitive theory (SCT) [43–45]. Adult learning theory posits that people prefer to learn based on real-life problems, by setting realistic goals, listening to their peers, and experiencing success when they experiment with improvement efforts. STUN Alcohol Use Now’s practice facilitation adheres to the preferences of the targeted adult learners (clinicians and practice staff) in primary care practices (Table 1).

**Table 1:** STUN Alcohol Use Now’s hypothesized influence

Adult learners learn…	STUN Alcohol Use Now approach
Based on real-life problems	Practice facilitation, expert consultation, and training modules will incorporate real problems
By setting realistic goals	Practice facilitation, expert consultation, and training modules will emphasize realistic goals
By listening to their peers	Practice facilitation, expert consultation, and training modules will incorporate peers in their stories
By experiencing success	Use of EHR reports, run charts, and positive reinforcement by facilitators

### Data and safety monitoring

Our Data Safety and Monitoring Plan (DSMP), commensurate with the low degree of risk involved in participation, will focus on monitoring and minimizing risks to participating practices, careful monitoring of the study’s progress, protecting the confidential medical and personal information of subjects, and ensuring the validity and integrity of the collected data.

The principal investigator will be responsible for carrying out the DSMP. Key personnel involved with the logistics of practice enrollment, participation, and follow-up (e.g., the overall project manager, AHEC project manager, and data manager) will meet regularly (at least quarterly) to review, among other things, progress on accrual, implementation, data collection, and adverse events. Many of these individuals, along with the study investigators, will meet weekly to address ongoing study issues including, among other things, progress on accrual (practice recruitment and retention), follow-up, and adverse events. The project manager will then prepare the needed reports (Table 2) for the PI.

**Table 2:** Data and Safety Monitoring Plan

Data type and description	Frequency of review
Practice recruitment and retention	Weekly, with monthly summaries for PI including a graphic of projected vs. actual
Study performance (via Practice Facilitator contact logs, we will capture number and types of personnel working with practices to support implementation, number, and type of interactions between project staff/consultants and practices, number of practices reached by the implementation, number of clinicians engaged, and percentage of PF contacts with a practice that are in-person)	Monthly summary to the PI All significant protocol deviations will be reported to the IRB during the annual review process
Implementation effectiveness (assessed via required measures.)	A monthly summary, including run charts, will be prepared from uploaded encrypted and secure data submitted from the practices to our database
Risks to participating practices (practice facilitators will be trained to monitor the time commitment of the practice to the project and to note any disruption in practice workflow or functions)	Monthly summary to the PI
Adverse events (AEs), such as breach of confidentiality	Practice facilitators will be trained to identify and report AEs as they occur to the AHEC project manager and overall project manager. The PI will be responsible for reporting AEs, as they occur, to the IRB, using UNC IRB definitions, standards, and forms
Stopping rules regarding benefits and harms	Not applicable to the study
Stopping rules regarding statistical power	Not applicable to the study

Annually, the PI will prepare a written report addressing the study’s progress and safety, which will be provided to the IRB as part of the annual renewal submission.

AHEC technical support personnel will assist participating practices with producing the discrete EHR data fields necessary to accrue the measures and reliably transfer them for processing. The practice facilitators will work with practice teams to create workflows that assure data entry into appropriate fields. Secure data will be uploaded to the project database on a monthly basis via an online system we can monitor for timeliness and completeness of data entry. The regular data monitoring during this study will allow us to identify missing data and, when feasible, obtain this information through phone calls or email follow-up. Where EHRs of small practices have limited capability to produce reports, we will provide web-based data entry tools and registries instead. The PFs will monitor, remind, and encourage the practices to complete surveys.

Project data is housed at the parent research center’s dedicated research computing servers for the analysis of large-scale research datasets. Access to these servers requires two-factor authentication, a restricted VPN connection, and is limited to specific programmers authorized by the center’s senior management. Once connected to the system, each programmer can access only data needed for their specific project, by way of operating-system-level security groups. In addition to these programmers, data is accessed by study personnel who have completed all required security and privacy training.

To minimize risks, all efforts will be made to collect data in a manner that protects a person’s right to confidential participation. Survey links that are sent, after the consent, to practice staff via email will remind staff that they may be completed at a time and place of their own choice. They also will be offered the option to complete the questions by phone if more comfortable for them. Programmers and research staff who work with sensitive data are required to complete appropriate HIPAA training with periodic updates, complete and comply with all institution training, Security Policies, and provisions.

### Constructs and measures

#### Implementation support

Using practice facilitation contact logs, we will capture the number and type of interactions between project staff/consultants and practices, the number of practices reached by the implementation, and the number of clinicians engaged.

#### Practice capacity for QI

Practice capacity for QI encompasses 2 constructs, change process capability and adaptive reserve. We will assess the former using the 32-item Change Process Capacity Questionnaire [46] and the latter using the 3-item adaptive reserve (i.e., capacity for change) scale [47, 48]. Both the CPCQ and the adaptive reserve scale exhibit reliability and known-group validity [46, 49].

#### Organizational readiness for change

Organizational readiness for change refers to the extent to which organizational members are psychologically and behaviorally prepared to implement organizational change [50–52]. We will measure readiness using the 12-item Organizational Readiness for Implementing Change (ORIC) scale [50] ORIC has demonstrated reliability, content validity, structural validity, structural invariance, and known-groups validity [50].

#### Implementation policies and practices

Implementation policies and practices are the strategies that an organization puts into place to support innovation use [53, 54]. We will measure these with the Key Driver Implementation Scale (KDIS), which uses a 5-point, behaviorally anchored, ordinal scale that covers multiple fundamental drivers of improvement.

#### Implementation climate

Implementation climate refers to organizational members’ “shared summary perception of the extent to which their use of a specific innovation is rewarded, supported, and expected within their organization” [55]. We will measure implementation climate using a 6-item scale with demonstrated reliability, structural validity, structural invariance, known-groups validity, and predictive validity.

#### Implementation effectiveness

Implementation effectiveness refers to the consistency and quality of innovation use [53]. Implementation effectiveness is conceived here as an organization-level construct describing organizational members’ pooled consistency and quality of innovation use (i.e., evidence-based screening, counseling, referrals, and pharmacotherapy) [53, 56, 57]. We will assess implementation effectiveness using the measures in Table 3.

**Table 3:** Implementation effectiveness measures

Measure	Description
# and % of patients screened	Number and percentage of adult patients with documentation of screening for unhealthy alcohol use with the screening questions recommended by NIAAA^a^
# and % of patients with a positive screen	Number and percentage of adult patients with a positive initial screen
# and % of patients completing the AUDIT	Number and percentage of those with a positive initial screen^a^ who go on to complete the 10-question AUDIT (the next step in assessment after an initial positive screen)
# and % of patients who receive brief counseling	Number and percentage of adult patients with documentation of brief counseling for risky drinking, when indicated/appropriate
# and % of patients who have AUD	Number and percentage of adult patients with documented ICD diagnoses of AUD
# and % of patients who receive pharmacotherapy for AUD	Number and percentage of adult patients with AUD who receive evidence-based pharmacotherapy with naltrexone, acamprosate, disulfiram, or topiramate^b^
# and % of patients referred to specialty care for AUD	Number and percentage of adult patients with AUD referred to specialty care (e.g., psychiatry, CBT, motivational enhancement therapy, 12-step programs)

*AUD* alcohol use disorder, *AUDIT* alcohol use disorders identification test, *CBT* cognitive behavioral therapy, *ICD* International Classification of Diseases, *NIAAA* National Institute on Alcohol Abuse and Alcoholism

^a^Do you sometimes drink beer, wine, or other alcoholic beverages? (If yes) How many times in the past year have you had 5 or more (for men 64 and younger)/4 or more (for women of any age, and men 65 and older) drinks in a day? [6] Response of 1 or more is a positive initial screen

^b^Topiramate is not FDA-approved for AUD, but it has been shown to be beneficial (e.g., for reducing heavy drinking days) [14]

#### Telehealth acceptability

Telehealth acceptability for those practices provided with telehealth services will be measured using a brief survey based on the Technology Acceptance Model [58, 59].

### Data collection

Table 4 outlines data collection, measures, sources, and timing for each construct.

**Table 4:** Theoretical constructs, measures, data sources, and data collection timing

Construct	Measure	Source	Timing
Implementation support	Frequency, duration, mode, and purpose of practice contacts	PF contact logs	I
Practice capacity for QI	Change Process Capacity Questionnaire (CPCQ) Adaptive Reserve Questionnaire	Provider/staff survey	B, E, F
Organizational readiness	Organizational Readiness for Change Questionnaire (ORIC)	Provider/staff survey	B
Implementation policies and practices	Key Driver Implementation Scale (KDIS) and type and quantity of strategies implemented	PF ratings	I
Implementation climate	Implementation Climate Questionnaire	Provider/staff survey	E, F
Implementation effectiveness	Number and % of patients in the target population who are screened for unhealthy alcohol use, screen positive, receive brief counseling, have AUD, receive pharmacotherapy, referred for AUD	Data forms on the website, chart review, or direct from EHR	B, I, E, F
Contextual factors	Practice characteristics, patient population, EHR capabilities	Provider/staff survey	B, I, E, F
Telehealth acceptability	Acceptability to practice; satisfaction with workflow and quality	Provider/staff survey	E, F

*PF* practice facilitator, *QI* quality improvement, *EHR* electronic health record, *AUD* alcohol use disorder, Timing: *B* baseline, *I* intervention (weekly/monthly), *E* end of the intervention, *F* 6-month post-intervention (i.e., 18 months after baseline)

### Practice facilitator contact logs

To capture *implementation support*, practice facilitators will record the date, duration, and mode of each practice contact. We will use these data to measure the level of implementation support provided to each practice.

### Provider/staff survey

We will survey providers and staff to assess practice capacity for QI at baseline, end-of-intervention (12 months after baseline), and 6 months post-intervention (18 months after baseline); organizational readiness to implement change at baseline; and implementation climate at end-of-intervention and 6 months post-intervention. To document *contextual factors*, surveys will also capture relevant practice characteristics, general patient population and demographics, and EHR capabilities. The survey sample at each time point will consist of 3–5 providers and staff members depending on practice size.

### Practice facilitator ratings of practices’ progress

Practice facilitators will assess practices’ progress in implementing clinical practice and organizational changes to support improvement in screening, brief intervention, and referral to treatment (SBIRT) (implementation policies and practices) at monthly intervals. Ratings will focus on the extent to which practices have implemented multiple key drivers of improved SBIRT provision and level of leadership and team engagement.

### Chart review

To assess screening rates at baseline, practice facilitators will assist practice staff in querying their EHR or conducting a chart review to determine the number and proportion of patients who have had evidence-based screening over the previous 2 years.

### EHR/informatics

Practices will obtain performance data on the implementation effectiveness (uptake) from their EHRs or by recording the data in a registry, the creation of which the practice facilitators and project team will guide. These data will be entered as aggregate counts and percentages for each practice (no protected health information [PHI] will be included) in a dedicated, online tool that feeds into the project database. The implementation effectiveness measures will be collected at baseline, during the intervention, at the end of the intervention, and post-intervention (6 month post-intervention follow-up, i.e., 18 months after baseline).

### Data analysis

#### Aim 1: Evaluate the effect of primary care practice facilitation on uptake of evidence-based SBI

Our primary hypotheses are that practice facilitation will increase the number and percentage of patients in a practice who are: (a) screened for unhealthy alcohol use, (b) identified to have unhealthy alcohol use, and (c) provided with brief counseling. Our secondary hypothesis is that practice capacity for QI, organizational readiness to implement change, implementation climate, and contextual factors will moderate the effect of primary care practice facilitation on the use of evidence-based SBI for unhealthy alcohol use.

##### Statistical analysis

For each practice, percent screened for unhealthy alcohol use will be computed using all data available from the baseline (henceforth “baseline”) as well as in each of the two quarters up to 6 months. 95% confidence intervals will be calculated with the percentages compared using repeated measures ANOVA for an overall test of change following practice facilitation, as well as for differences in percentages for each of the post-facilitation quarters relative to pre-baseline. The changes in percent screened will be evaluated with paired *t* tests looking at differences between each pair of quarters at alpha=.05. The primary analysis will be based on a comparison of the baseline versus the 2nd quarter following practice facilitation, where the impact of practice facilitation is likely to be greatest. The repeated measures ANOVA will allow us to potentially detect a delay in effectiveness. Similar analyses will be conducted using percent identified with unhealthy alcohol use and for the percent provided with counseling, based on the data from baseline and up to 6 months after practice facilitation initiation. The analyses will be redone adjusting for the calendar time period to control for potential changes occurring naturally over time. As a secondary analysis, we will examine proportion trajectories using a mixed modeling approach, iteratively testing linear, quadratic, and cubic time functions (Level 1); interacting those time functions with intervention conditions (Level 2); and controlling for key covariates (e.g., clinic size). Relative model fit statistics (e.g., Akaike information criterion [AIC]) will be used to inform the selection of the final model.

We will examine the effects of practice characteristics, such as organizational readiness and implementation environment on the impact of practice facilitation. Descriptive statistics will be calculated for practice-level variables. Linear regression models will be fit to practice level change scores based on differences in the two quarters after practice facilitation compared to the baseline percentage. Generalized estimating equations will be used to fit models simultaneously using the two change scores. Each moderating variable will be screened in univariate models, with significant variables entered in a multivariable model. AIC will be used to choose the final model.

Secondary analyses will consider quarterly data collected after 6 months to the end of follow-up at 12 months, and to the post-intervention follow-up, to evaluate the effect of practice facilitation as well as the effect of telehealth combined with practice facilitation. This will involve analyses similar to those above in which all practices that are randomized to no telehealth are grouped together, while higher-performing practices at 6 months randomized to telehealth (but not receiving telehealth) and lower performing practices randomized to telehealth constitute a second group. Change scores will be calculated from baseline. Average percent improvements from baseline between the two groups will be compared at each quarter after 6 months using two-sample *t* tests, with an overall test based on repeated measures ANOVA. Adjustment for practice-level characteristics will be explored using GEE. Survey data collected from multiple clinic staff/health care providers will be analyzed using a mixed modeling approach, with a person (Level 1) nested within the clinic (Level 2)—where appropriate, three-level models with repeated measures (Level 1), within person (Level 2), and within clinic (Level 3) will be employed when considering time effects for these data.

Patterns of missing data will be assessed descriptively. Associations between practice level covariates and missingness will be explored, with differences formally tested using logistic regression models for binary missingness variables. Inverse weighting based on missingness probabilities will be employed to adjust for the effects of practice level covariates on missingness, where appropriate. Primary analyses will follow the intention to treat principles.

The main outcomes are being measured at the practice level (e.g., did the practice increase their rates of screening for unhealthy alcohol use), not at the individual patient level, and the unit of inference is the practice (not the individual patient). Therefore, the trial was not designed as a cluster trial and there is not a need to account for clustering; trials focused on outcomes measured at the group/practice level can be regarded as standard clinical trials with respect to the estimation of sample size and analysis approach [60, 61].

##### Statistical power

We estimate that a very low percentage (<5%) currently receives recommended screening. On average, based on our experience and prior evaluations we expect about 45% of patients within each practice to receive screening following practice facilitation, by 6 months. For unhealthy alcohol use, preliminary data suggest that about 25% of those screened will have unhealthy alcohol use identified (roughly 7.5% of all adults served by the practice). Conservatively, if there is a 10% increase in the percentage (of either screening or detection of unhealthy alcohol use from baseline to 6 months) on average across practices, with a standard deviation in the percentage improvement of 10%, then a one-sample *t* test at level 0.05 has at least 80% power to detect that improvement with sample size 34. We expect that the increase in the percentage of adults screened would be higher than 10% and that fewer practices would be needed to detect a significant increase; our prior work (when our intervention was less developed) showed an increase of more than 30% over 6 months [19–21]. Thus, the proposed design has very good power to detect realistic effect sizes for improved screening and detection of unhealthy alcohol use, even with considerable dropout and/or missing data (even more than 30% missingness).

#### Aim 2: Evaluate the effect of practice facilitation on uptake of evidence-based counseling and pharmacotherapy for AUD

Our hypotheses are that practice facilitation will increase the number and percentage of patients in a practice who are (a) identified to have AUD, (b) provided with pharmacotherapy for AUD, and (c) referred to specialty care for AUD.

##### Statistical analysis

As in Aim 1, for each practice, the percent identified with AUD will be computed using all data available. 95% confidence intervals will be calculated with the percentages compared using repeated measures ANOVA for an overall test of change following practice facilitation, with paired *t* tests looking at differences between each pair of quarters. The primary analysis will be based on a comparison of the baseline and the 2nd quarter following practice facilitation. Similar analyses will be conducted using percent-provided pharmacotherapy and referred to specialty care for AUD, based on the data from baseline and up to 6 months after practice facilitation initiation. Additional secondary analyses will consider quarterly data collected after 6 months from baseline to the end of follow-up at 12 months, and to the post-intervention follow-up (18 months), using the intent to treat framework described for Aim 1. Missingness for Aim 2 endpoints will be assessed as in Aim 1. Mixed modeling will be used to compute trajectory models as described for Aim 1.

##### Statistical power

Detection of previously unknown AUD via screening in primary care is relatively rare, on the order of 1% among adults with no history of AUD. Assuming 45% of adults are screened over 6 months, we would expect an increase of roughly 0.45% from baseline following practice facilitation. Among those with AUD, we anticipate a relatively large increase in pharmacotherapy for AUD, from 0 to 33% on average. To detect an increase of 0.45% in AUD detection on average with a standard deviation of 1% using a one-sample *t* test at level 0.05, sample sizes of 39 and 52 give 80% and 90% power to detect such differences. The increase in pharmacotherapy for AUD for those identified as having AUD is quite large and the one-sample *t* tests have very large power to detect such improvements.

#### Aim 3: For practices with slower uptake, evaluate the effect of telehealth services on the use of evidence-based (a) SBI for unhealthy alcohol use and (b) counseling and pharmacotherapy for AUD

Our primary hypotheses are that, compared with continued practice facilitation, practices randomized to the provision of telehealth services will increase the number and percentage of patients who are (a) provided with brief counseling for unhealthy alcohol use, (b) provided with pharmacotherapy for AUD, and (c) referred to specialty care for AUD. Our secondary hypothesis is that telehealth services for counseling will be acceptable to small to medium-size primary care practices.

##### Statistical analysis

Practices in the lower 50th percentile at 6 months will be randomized to telehealth or not. The primary analysis will be an ITT analysis based on a comparison of changes in counseling, pharmacotherapy, and specialty care from 6 months to 12 months (end-of-intervention). Such changes will be calculated based on a comparison of quarterly percentages, where 3–6 months serves as a baseline and 6–9 and 9–12 months as the post-randomization quarters. Note that for this aim, the 6-month baseline differs from the baseline in Aims 1 and 2. Average percent changes and 95% confidence intervals will be computed for SBI and pharmacotherapy in the two intervention arms for 6–9 and 9–12 months. The two arms will be compared using repeated measures ANOVA and two-sample *t* tests at each quarter at level .05. The main focus is on the changes from 3–6 months to 9–12 months, where the impact of telehealth is likely to be greatest. The repeated measures ANOVA will allow us to potentially detect increased impact over time. Secondary analyses will assess the post-intervention follow-up (focusing on change from 3–6 to 15–18 months) and acceptability of telehealth. Acceptability percentage will be calculated across practices, along with 95% confidence intervals. Mixed modeling will be used to assess the impact of practice-level characteristics on acceptability on the part of staff/healthcare providers.

##### Statistical power

The focus is on detecting differences in the changes from 6 to 12 months for practice facilitation + telehealth versus practice facilitation without telehealth. Differences will be tested at level .05 using two-sample *t* tests based on an ITT analysis of percentage changes in the two randomized groups. Assuming that the standard deviation of the percent changes is .15 in each group (and a between-group difference of 20%), a sample size of 9 per group gives 80% power and a sample size of 12 gives 90% power. The resulting effect sizes are moderate in size and consistent with previous results.

#### Aim 4: Evaluate the effect of practice facilitation on the implementation of clinical practice and office systems changes to improve evidence-based SBI and pharmacotherapy

Our primary hypothesis is that practice facilitation will increase the implementation of clinical practice and office systems changes to improve evidence-based SBI and pharmacotherapy. Our secondary hypotheses are that (a) practice capacity for QI, organizational readiness to implement change, and contextual factors will moderate the effect of practice facilitation on the implementation of clinical practice and office systems changes and (b) provision of telehealth services will increase implementation of clinical practice and office systems changes among practices with slower uptake.

##### Statistical analysis

This aim is structured to explore the impact of practice facilitation and telehealth on the implementation of clinical practice and office systems changes. The analysis plan mirrors that in Aims 1 and 2. The first set of analyses is based on outcomes from the first two quarters from baseline to 6 months in which the effect of practice facilitation is explored, independently of telehealth. The second set of analyses compares the effect of practice facilitation with and without telehealth using quarterly data from 6 to 12 months. Moderating effects of practice-level characteristics will be explored in both sets of analyses.

##### Statistical power

These power calculations are similar to those for Aims 1 and 2 which focused on the impact of practice facilitation. Here, the primary endpoint is detecting a difference in KDIS score from baseline, which is evaluated using a one-sample *t* test. A meaningful improvement would be 1 unit. With standard deviation of KDIS differences equaling 3 (a conservative assumption), sample sizes of 70 and 95 give 80% and 90% power based on a .05 level test.

### Dissemination plan

The overall goal of our dissemination plan is to inform all stakeholders including patients, providers, payers, and government agencies about the process and findings of STUN Alcohol Use Now. The main message will be that small to medium-size primary care practices are partnering with NC AHEC to rapidly improve the implementation of SBI and MAT for unhealthy alcohol use. These efforts are expected to prevent deaths from unhealthy alcohol use as well as morbidity from the many adverse health consequences. We will commit to working with AHRQ and its contractors in disseminating information about the project, such as through the AHRQ annual meeting and AHRQ publications. We will fully inform AHRQ and its designated contractors regarding implementation results and status of outcomes over time. All presentations and publications derived from STUN Alcohol Use Now will also be made available to AHRQ and its contractors.

By involving our stakeholders, we have already enlisted key people and organizations with the wherewithal and enthusiasm to support the dissemination of this project. We have begun to work closely with the NC AHEC Practice Support Program, the NCHQA, the NC Academy of Family Practice, Carolina Partners in Mental HealthCare, UNC’s Alcohol and Substance Abuse Program, UNC’s Virtual Care Center, and UNC-CH’s practice-based research network, NCNet. AHEC has already informed their networks about the possibility of this work and the vast potential for reducing unhealthy alcohol use and improving outcomes using this proposal as a nidus. Additional specific organizations interested in research for unhealthy alcohol use that we intend to engage include the nascent accountable care organizations in NC and beyond, the NC Chapter of the American College of Physicians, the American Public Health Association, BCBS NC, the Society of General Internal Medicine, the National AHEC Organization, and Academy Health.

Once informed of the award, NC AHEC will be able to activate many associated practices before the actual funding using teams that already touch hundreds of primary care practices. The Steering Committee, that will include patient advocates yet to be named, will be regularly briefed on the process and results during every quarterly meeting. As we document evidence of process improvement, practice acceptance, and improved outcomes, we will provide the committee members with materials to distribute to their constituents without delay. The AHEC Practice Support Program remains closely connected to the NC Office of Health Benefits and Division of Public Health and often shares improvement results of ongoing efforts with these state agencies.

As we have done in the past, we will use community forums, local media outlets, scientific meetings, and publications to disseminate findings. AHEC is developing a presence on social media that could be used to disseminate unhealthy alcohol use awareness to new audiences. The practices involved in the actual study represent only a fraction of AHEC practices. The larger network provides a ripe environment for dissemination.

## Discussion

This study will assess whether primary care practice facilitation can achieve dissemination and implementation of evidence-based screening and services for unhealthy alcohol use and will help clarify what practice support activities affect the uptake of recommended screening, brief interventions, and referral and treatment for unhealthy alcohol use. Additionally, this comprehensive evaluation will determine if there are differential practice outcomes based on the level of readiness for change and external factors in the practice environment.

Practice facilitators are trained “to meet the practice where they are” and adjust the approach to address barriers and reignite progress. Our experimental question is whether facilitation and other multi-modal supports can achieve fast initiation, spread, and improvement, especially since most practices have done little with systematic alcohol screening and treatment despite years of evidence supporting this work. We will set up the intervention process with the urgency of the task, understanding that the judgment of the facilitators may sometimes have to intervene and recalibrate the pace that a practice can maintain.

A strength of the NC AHEC practice support program is the ability of practice facilitators to assess what a practice needs to implement and to excel in process and outcome improvement [62, 63]. We expect that practice facilitation and embedded telehealth may be effective because they each address key barriers. Barriers can include reticent providers and financial stressors. More subtle factors such as lack of trust, lack of knowledge, fear of change, and disbelief in the evidence can also pose obstacles to enacting organizational change. Specific to SBI for unhealthy alcohol use, competing priorities and limited time pose key barriers for primary care providers [19–21]. The initial screening itself can be done quickly, but we found that providers require an estimated 5–10 min to perform the screening-related assessment when a patient has positive screening results; additional time and visits are required for counseling those with risky drinking behavior [9, 19, 20, 64]. Other barriers specific to unhealthy alcohol use include that many providers/staff/practices lack knowledge of intervention options or training in delivery, or lack familiarity with the rationale and tools for implementation [19, 20]. Further, it can be difficult to adapt EHRs to newer clinical measures. Finally, the small rural primary care practices in our study often have limited access to services (e.g., counseling for AUD) both within their practice and within the county.

Potential barriers to practice study enrollment include fear of financial risk, fully dedicating practice resources to other new activities such as Medicaid reform within the state or Accountable Care Organization participation, as well as perceptions of staffing issues, competing priorities, and lack of time. The mitigation strategy for these barriers will be to align practice facilitation to help the practice achieve some of these goals and offering incentives. Also, AHEC serves as a facilitator as it maintains strong relationships with most targeted practices marked by recurring meetings and continuing education programs. AHEC also maintains strong relationships with leading hospitals and health systems, and in some cases contracted work with many of the health systems involved in practice acquisitions. AHEC also supports primary care residency programs from which many participating providers graduated. We will leverage these relationships to boost accrual and prevent attrition. Instead of bringing in an intervention that is foreign to the practices, STUN will work with a known entity (NC AHEC) that can promote implementation through existing relationships, processes, and personnel.

If successful, our intervention would better identify and significantly reduce the burden of disease from unhealthy alcohol use in participating clinics. The study will produce fundamentally important evidence about the effect of practice facilitation on uptake of evidence-based SBI for unhealthy alcohol use when delivered on a large scale to small- to medium-sized primary care practices. It will also generate scientific knowledge about whether telehealth services can improve the use of evidence-based screening and interventions for practices with slower uptake. The results of this rigorously conducted evaluation are expected to have a positive impact by supplying evidence on effective and efficient dissemination and implementation of evidence related to addressing unhealthy alcohol use into primary care practices.

### Trial status

At the time of manuscript completion, the trial is in the recruitment stage. Recruitment began in January 2020. This protocol is version 1, dated September 10, 2021. The end of the trial is expected in September 2023.

## Supplementary Information

**Additional file 1.**

**Additional file 2.**

**Additional file 3.**

## Data Availability

The full study protocol is available through this publication and through the ClinicalTrials.gov record. The results of the study will be provided in ClinicalTrials.gov, when available, and through the publication of findings. The resources for primary care practices and materials used for the practice facilitation intervention are available on the study website (https://stunalcoholusenow.org/).
