# Supplementary material for: The STUN (STop UNhealthy) Alcohol Use Now trial: study protocol for an adaptive randomized trial on dissemination and implementation of screening and management of unhealthy alcohol use in primary care
Source: Trials. 2021 Nov 16;22:810. doi: 10.1186/s13063-021-05641-7 (PMC8593635; doi:10.1186/s13063-021-05641-7)
Supplement: Supplementary file 2 — Additional file 2. [file 13063_2021_5641_MOESM2_ESM.docx]

**World Health Organization Trial Registration Data Set**

| **Item name** | **Item description** | **Response** |
| --- | --- | --- |
| Primary Registry and Trial Identifying Number | Name of Primary Registry, and the unique ID number assigned by the Primary Registry to this trial. | ClinicalTrials.gov NCT04317989 (page 3, line 62) |
| Date of Registration in Primary Registry | Date when trial was officially registered in the Primary Registry. | March 23, 2020 (page 3, line 62) |
| Secondary Identifying Numbers | Other identifiers besides the Trial Identifying Number allocated by the Primary Registry, if any. These include:   - The Universal Trial Number (UTN) - Identifiers assigned by the sponsor (record Sponsor name and Sponsor-issued trial number (e.g. protocol number)) - Other trial registration numbers issued by other Registries (both Primary and Partner Registries in the WHO Registry Network, and other registries) - Identifiers issued by funding bodies, collaborative research groups, regulatory authorities, ethics committees / institutional review boards, etc.   All secondary identifiers will have 2 elements: an identifier for the issuing authority (e.g. NCT, ISRCTN, ACTRN) plus a number. There is no limit to the number of secondary identifiers that can be provided. | Agency for Healthcare Research & Quality (1R18HS027078-01)  UNC IRB Study # 19-1853 |
| Source(s) of Monetary or Material Support | Major source(s) of monetary or material support for the trial (e.g. funding agency, foundation, company, institution). | Agency for Healthcare Research & Quality |
| Primary Sponsor | The individual, organization, group or other legal entity which takes responsibility for initiating, managing and/or financing a study. The Primary Sponsor is responsible for ensuring that the trial is properly registered. The Primary Sponsor may or may not be the main funder. | Agency for Healthcare Research & Quality |
| Secondary Sponsor(s) | Additional individuals, organizations or other legal persons, if any, that have agreed with the primary sponsor to take on responsibilities of sponsorship.  A secondary sponsor may have agreed to:   - take on all the responsibilities of sponsorship jointly with the primary sponsor; or - form a group with the Primary Sponsor in which the responsibilities of sponsorship are allocated among the members of the group; or - act as the Primary Sponsor’s legal representative in relation to some or all of the trial sites. | University of North Carolina, Chapel Hill |
| Contact for Public Queries | Email address, telephone number and postal address of the contact who will respond to general queries, including information about current recruitment status.  “Note: The information provided in here is functional and not personal, it is recommended to provide institutional and not personal information. By providing this information the registrant consents that the information provided can or may be published on a public website. Once provided the information cannot be redacted or anonymized as a result of new privacy legislation such as the European General Data Protection Regulation (GDPR)”. | Daniel E. Jonas, MD, MPH  Division of General internal Medicine  College of Medicine  The Ohio State University  2050 Kenny Rd., Columbus OH 43221  (614) 366-8517  [Daniel.Jonas@osumc.edu](mailto:Daniel.Jonas@osumc.edu) |
| Contact for Scientific Queries | There must be clearly assigned responsibility for scientific leadership to a named Principal Investigator. The PI may delegate responsibility for dealing with scientific enquiries to a scientific contact for the trial. This scientific contact will be listed in addition to the PI.  “Note: The information provided in here is functional and not personal, it is recommended to provide institutional and not personal information. By providing this information the registrant consents that the information provided can or may be published on a public website. Once provided the information cannot be redacted or anonymized as a result of new privacy legislation such as the European General Data Protection Regulation (GDPR)”.  The contact for scientific queries must include:   - Name and title, email address, telephone number, postal address and affiliation of the Principal Investigator, and; - Email address, telephone number, postal address and affiliation of the contact for scientific queries about the trial (if applicable). The details for the scientific contact may be generic (that is, there does not need to be a named individual): e.g. a generic email address for research team members qualified to answer scientific queries. | Daniel E. Jonas, MD, MPH  Division of General internal Medicine  College of Medicine  The Ohio State University  2050 Kenny Rd., Columbus OH 43221  (614) 366-8517  [Daniel.Jonas@osumc.edu](mailto:Daniel.Jonas@osumc.edu) |
| Public Title | Title intended for the lay public in easily understood language. | STUN (STop UNhealthy) Alcohol Use Now! |
| Scientific Title | Scientific title of the study as it appears in the protocol submitted for funding and ethical review. Include trial acronym if available. | The STUN (STop UNhealthy) Alcohol Use Now! Project: Using Practice Facilitation to Disseminate and Implement Patient-Centered Outcomes Research (PCOR) Evidence on Screening and Management of Unhealthy Alcohol Use in Primary Care |
| Countries of Recruitment | The countries from which participants will be, are intended to be, or have been recruited at the time of registration. | United States |
| Health Condition(s) or Problem(s) Studied | Primary health condition(s) or problem(s) studied (e.g., depression, breast cancer, medication error).  If the study is conducted in healthy human volunteers belonging to the target population of the intervention (e.g. preventive or screening interventions), enter the particular health condition(s) or problem(s) being prevented. | Unhealthy alcohol use |
| Intervention(s) | For each arm of the trial record a brief intervention name plus an intervention description.  Intervention Name: For drugs use generic name; for other types of interventions provide a brief descriptive name.   - For investigational new drugs that do not yet have a generic name, a chemical name, company code or serial number may be used on a temporary basis. As soon as the generic name has been established, update the associated registered records accordingly. - For non-drug intervention types, provide an intervention name with sufficient detail so that it can be distinguished from other similar interventions.   Intervention Description: Must be sufficiently detailed for it to be possible to distinguish between the arms of a study (e.g. comparison of different dosages of drug) and/or among similar interventions (e.g. comparison of multiple implantable cardiac defibrillators). For example, interventions involving drugs may include dosage form, dosage, frequency and duration.  If the intervention is one or more drugs then use the International Non-Proprietary Name for each drug if possible (not brand/trade names). For an unregistered drug, the generic name, chemical name, or company serial number is acceptable.  If the intervention consists of several separate treatments, list them all in one line separated by commas (e.g. "low-fat diet, exercise").  For controlled trials, the identity of the control arm should be clear. The control intervention(s) is/are the interventions against which the study intervention is evaluated (e.g. placebo, no treatment, active control). If an active control is used, be sure to enter in the name(s) of that intervention, or enter "placebo" or "no treatment" as applicable. For each intervention, describe other intervention details as applicable (dose, duration, mode of administration, etc). | All enrolled practices receive 6 months of the practice facilitation intervention. Practices with performance in the upper 50th percentile (based on primary outcome measures at that time) will receive practice facilitation for the duration of the intervention period. Practices with performance in the lower 50^th^ percentile will be randomized to 2 arms. Of those practices randomized, one arm will receive continued practice facilitation plus embedded telehealth services related to interventions for unhealthy alcohol use. The other arm will receive ongoing practice facilitation for the duration of the intervention period (but will not receive embedded telehealth services).  Practice Facilitation:   - Implementing evidence-based protocols and the use of clinical algorithms (for screening, counseling, referral, and MAT) to engage the entire clinical team in a high standard delivery of care. - Promoting a strong use of decision support tools and templates to support the practice workflow. - Optimizing the use of the electronic health record (EHR) to pull clinical data on a monthly basis to guide the change process. - Developing patient registries (e.g., for those identified to have AUD) to identify needed care - Proactive, team-based care with assigned roles and responsibilities to prepare the clinical team to develop needed care and engage patients throughout the entire visit process. - Enhancing the understanding of available counseling and referral resources to ensure that practices are confident that they have appropriate evidence-based intervention options.   Embedded Telehealth Services:  Establishment within participating practices of video conferencing infrastructure where patients identified as having unhealthy alcohol use may receive counseling from remotely located providers. |
| Key Inclusion and Exclusion Criteria | Inclusion and exclusion criteria for participant selection, including age and sex. Other selection criteria may relate to clinical diagnosis and co-morbid conditions; exclusion criteria are often used to ensure patient safety.  If the study is conducted in healthy human volunteers not belonging to the target population (e.g. a preliminary safety study), enter "healthy human volunteer". | The study is enrolling at the practice level, rather than the patient level. Screening and interventions for unhealthy alcohol use are recommended services in primary care for adult patients (18 years and older).  Practice inclusion criteria: Small-to-medium sized primary care practices (10 or fewer providers) in North Carolina.  Practice exclusion criteria: practices with fewer than 100 adult patients (18+ years) or more than 10 providers; practices unwilling to implement evidence-based screening and management of patients with unhealthy alcohol use. |
| Study Type | Study type consists of:   - Type of study (interventional or observational) - Study design including: - Method of allocation (randomized/non-randomized)   - Masking (is masking used and, if so, who is masked)   - Assignment (single arm, parallel, crossover or factorial)   - Purpose - Phase (if applicable)   For randomized trials: the allocation concealment mechanism and sequence generation will be documented. | Type of study: interventional  Allocation: adaptive randomized  Assignment: sequential  Masking: none  Purpose: to evaluate the effect of primary care PF and embedded telehealth services on evidence-based screening, counseling, and Medication-Assisted Treatment (MAT) |
| Date of First Enrollment | Anticipated or actual date of enrollment of the first participant. | January 20, 2020 |
| Sample Size | Sample Size consists of:   - Number of participants that the trial plans to enroll in total. - Number of participants that the trial has enrolled. | - For the main aim, sample size calculations determined that 35 practices are needed to detect a 10% increase in the main outcome (percent screened for unhealthy alcohol use) over 6 months (which is a fairly small increase). Fewer practices would be needed if the magnitude of the increase in screening after intervention is larger than 10%, as we expect it to be based on our prior work. - As of March 2021, 18 practices have officially enrolled. |
| Recruitment Status | Recruitment status of this trial:   - Pending: participants are not yet being recruited or enrolled at any site - Recruiting: participants are currently being recruited and enrolled - Suspended: there is a temporary halt in recruitment and enrolment - Complete: participants are no longer being recruited or enrolled - Other | Recruiting |
| Primary Outcome(s) | Outcomes are events, variables, or experiences that are measured because it is believed that they may be influenced by the intervention.  The Primary Outcome should be the outcome used in sample size calculations, or the main outcome(s) used to determine the effects of the intervention(s). Most trials should have only one primary outcome.  For each primary outcome provide:   - The name of the outcome (do not use abbreviations) - The metric or method of measurement used (be as specific as possible) - The timepoint(s) of primary interest   Example:  Outcome Name: Depression  Metric/method of measurement: Beck Depression Score  Timepoint: 18 weeks following end of treatment | Outcome name: Number of adults screened for unhealthy alcohol use  Metric/method of measurement: Number of patients aged 18 or older who were screened with a validated tool for unhealthy alcohol use, ascertained via Electronic Health Record or practice registry  Time points: 6 months (primary outcome), 12 months, 18 months  Outcome name: Percent of adults screened for unhealthy alcohol use  Metric/method of measurement: Percent of patients aged 18 or older who were screened with a validated tool for unhealthy alcohol use, ascertained via Electronic Health Record or practice registry  Timepoints: 6 months (primary outcome), 12 months, 18 months |
| Key Secondary Outcomes | Secondary outcomes are outcomes which are of secondary interest or that are measured at timepoints of secondary interest. A secondary outcome may involve the same event, variable, or experience as the primary outcome, but measured at timepoints other than those of primary interest.  As for primary outcomes, for each secondary outcome provide:   - The name of the outcome (do not use abbreviations) - The metric or method of measurement used (be as specific as possible) - The timepoint(s) of interest | - Outcome name: Number of adults with a positive screen for unhealthy alcohol use   Metric/method of measurement: Of patients aged 18 or older who were screened for unhealthy alcohol use, number who had a positive initial screening result, ascertained via the medical record or practice registry  Timepoints: 6 months, 12 months, 18 months   - Outcome name: Percent of adults with a positive screen for unhealthy alcohol use   Metric/method of measurement: Of patients aged 18 or older who were screened for unhealthy alcohol use, percentage who had a positive initial screening result, ascertained via the medical record or practice registry  Timepoints: 6 months, 12 months, 18 months   - Outcome name: Number of adults provided with brief counseling for risky drinking   Metric/method of measurement: Of adult patients who were identified as having unhealthy alcohol use, number who received brief counseling (based on documentation in the medical record)  Timepoints: 6 months, 12 months, 18 months   - Outcome name: Percent of adults provided with brief counseling for risky drinking   Metric/method of measurement: Of adult patients who were identified as having unhealthy alcohol use, percentage who received brief counseling (based on documentation in the medical record)  Timepoints: 6 months, 12 months, 18 months   - Outcome name: Number of adults identified as having alcohol use disorder (AUD)   Metric/method of measurement: After screening, number of adult patients identified to have AUD (based on documented ICD diagnoses of AUD)  Timepoints: 6 months, 12 months, 18 months   - Outcome name: Percent of adults identified as having alcohol use disorder (AUD)   Metric/method of measurement: After screening, percentage of adult patients identified to have AUD (based on documented ICD diagnoses of AUD)  Timepoints: 6 months, 12 months, 18 months   - Outcome name: Number of adults prescribed pharmacotherapy for AUD   Metric/method of measurement: After screening, number of adult patients with AUD who receive evidence-based pharmacotherapy with naltrexone, acamprosate, disulfiram, or topiramate  Timepoints: 6 months, 12 months, 18 months   - Outcome name: Percent of adults prescribed pharmacotherapy for AUD   Metric/method of measurement: After screening, percentage of adult patients with AUD who receive evidence-based pharmacotherapy with naltrexone, acamprosate, disulfiram, or topiramate  Timepoints: 6 months, 12 months, 18 months   - Outcome name: Number of adults with AUD referred to specialty care for AUD   Metric/method of measurement: After screening, number of adult patients identified as having AUD who are referred to specialty care (e.g., psychiatry, CBT, motivational enhancement therapy, 12-step programs)  Timepoints: 6 months, 12 months, 18 months   - Outcome name: Percent of adults with AUD referred to specialty care for AUD   Metric/method of measurement: After screening, percentage of adult patients identified as having AUD who are referred to specialty care (e.g., psychiatry, CBT, motivational enhancement therapy, 12-step programs)  Timepoints: 6 months, 12 months, 18 months |
| Ethics Review | The ethics review process information of the trial record in the primary register database. It consists of:   - Status (possible values: Not approved, Approved, Not Available) - Date of approval - Name and contact details of Ethics committee(s) | Status: Approved  Date of Approval: Initial release 03/15/2020; last release 07/07/2020  Name and contact details of Ethics Committee: Non-Biomedical IRB, University of North Carolina at Chapel Hill. Phone: 919-966-3113 Email: [irb_compliance@unc.edu](mailto:irb_compliance@unc.edu) Address: 720 Martin Luther King Jr. Blvd., Bldg#385, Second Floor, Chapel Hill, NC 27599-7097 |
| Completion date | Date of study completion: The date on which the final data for a clinical study were collected (commonly referred to as, "last subject, last visit"). | N/A |
| Summary Results | It consists of:   - Date of posting of results summaries - Date of the first journal publication of results - URL hyperlink(s) related to results and publications - Baseline Characteristics: Data collected at the beginning of a clinical study for all participants and for each arm or comparison group. These data include demographics, such as age and sex, and study-specific measures. - Participant flow: Information to document the progress and numbers of research participants through each stage of a study in a flow diagram or tabular format. - Adverse events: An unfavorable change in the health of a participant, including abnormal laboratory findings, and all serious adverse events and deaths that happen during a clinical study or within a certain time period after the study has ended. This change may or may not be caused by the intervention being studied. - Outcome measures: A table of data for each primary and secondary outcome measure and their respective measurement of precision (eg a 95% confidence interval) by arm (that is, initial assignment of participants to arms or groups) or comparison group (that is, analysis groups), including the result(s) of scientifically appropriate statistical analyses that were performed on the outcome measure data, if any. - URL link to protocol file(s) with version and date - Brief summary | No results at this time |
| IPD sharing statement | Statement regarding the intended sharing of deidentified individual clinical trial participant-level data (IPD). Should indicate whether or not IPD will be shared, what IPD will be shared, when, by what mechanism, with whom and for what types of analyses. It consists of:   - Plan to share IPD (Yes, No) - Plan description | The study is not collecting individual-level participant data |
