# Supplementary material for: The STUN (STop UNhealthy) Alcohol Use Now trial: study protocol for an adaptive randomized trial on dissemination and implementation of screening and management of unhealthy alcohol use in primary care
Source: Trials. 2021 Nov 16;22:810. doi: 10.1186/s13063-021-05641-7 (PMC8593635; doi:10.1186/s13063-021-05641-7)
Supplement: Supplementary file 3 — Additional file 3. [file 13063_2021_5641_MOESM3_ESM.docx]

Your practice is participating in the STUN (STop Unhealthy) Alcohol Use Now study. Please review the concise summary and consent information below. By clicking on the individualized link at the bottom, you provide your consent to complete surveys as part of your practice’s participation.

CONCISE SUMMARY

The purpose of this research study is to examine whether practice facilitation, with support from quality improvement coaches, in small primary care practices leads to increases in screening for unhealthy alcohol use, identification of patients with unhealthy alcohol use, provision of brief counseling, and treatment and referral for patients identified with alcohol use disorder.

As part of the study, we are conducting online surveys to learn more about how practices like yours identify and manage unhealthy alcohol use among your patients. The surveys we are asking you to complete focus on your practice’s quality improvement experience, electronic health records capabilities, and general organizational characteristics. Participants will complete a longer survey at baseline (about 30 minutes to complete) and two shorter follow up surveys (less than 10 minutes to complete) over two years.

Participation in this research presents a very small risk of breach of confidentiality, which we will take all precautions to minimize.

If you are interested in learning more about this study, please continue to read below.

**Consent to Participate in a Research Study**

**Title of Study: Stop Unhealthy (STUN) Alcohol Use Now**

**Principal Investigator:** Daniel E. Jonas, MD, MPH

**Principal Investigator Department:** General Internal Medicine

**Principal Investigator Phone Number:** 919-966-2276 ext. 224

**Principal Investigator Email Address:** [daniel_jonas@med.unc.edu](mailto:daniel_jonas@med.unc.edu)

**Project Coordinator:** Colleen Barclay, MPH

**Project Coordinator Phone Number:** 919-966-5826

**Project Coordinator Email Address:** [cjb@unc.edu](mailto:cjb@unc.edu)

**Funding Source and/or Sponsor:** Agency for Healthcare Research and Quality (AHRQ)

**What are some general things you should know about research studies?** You are being asked to take part in a research study. To join is voluntary. You may choose not to participate, or you may withdraw your consent to be in the study, for any reason, without penalty.

**What is the purpose of this study?** The purpose is to examine whether practice facilitation, with support from quality improvement coaches, leads to increases in screening for unhealthy alcohol use, identification of patients with unhealthy alcohol use, provision of brief counseling, and treatment and referral for patients identified with alcohol use disorder.

**How many people will take part in this study?** If you decide to be in this study, you will be one of approximately 675 people completing surveys.

**What will happen if you take part in the study?** During this study, you will complete up to 3 online questionnaires over the course of 2 years.

**What are the possible benefits from being in this study?** Research is designed to benefit society by gaining new knowledge. Your practice may benefit from the support of quality improvement coaches, but you may not benefit personally.

**What are the possible risks or discomforts involved from being in this study?** We anticipate few risks in this study. There is a very small risk of breach of confidentiality and of experiencing some stress related to answering the survey questions.

**How will your privacy be protected? All efforts will be made to collect data in a manner that protects your right to confidential participation. Your responses will be stored in a secure database, with no identifying information connected to them.**

**What if you want to stop before your part in the study is complete?** You can withdraw from this study at any time, without penalty, and skip any question for any reason.

**Will you receive anything for being in this study? Will it cost anything?** After completing the baseline survey, you will receive a check or gift card for $80. For each of the follow up surveys, you will receive $30. There are no costs associated with being in the study.

**What if you have questions about this study?** You have the right to ask, and have answered, any questions you may have about this research. Contact the project coordinator or principal investigator listed above with any questions, complaints, or concerns you may have.

**What if you have questions about your rights as a research participant?** All research on human volunteers is reviewed by a committee that works to protect your rights and welfare. If you have questions or concerns, or if you would like to obtain information or offer input, please contact the Institutional Review Board at 919-966-3113 or by email to [IRB_subjects@unc.edu](mailto:IRB_subjects@unc.edu).

**By clicking “I consent” below, you provide your consent and you will be linked directly to a survey. Please try to complete the survey in one session. However, if you need more time, you can use the “I consent” link below to return to the survey in the future.**

**I consent** 
